# Reflecting on the Anthropocene: The Call for Deeper Transformations: *This article belongs to* Ambio*’s 50th Anniversary Collection. Theme: Anthropocene*

**DOI:** 10.1007/s13280-020-01468-9

**Published:** 2021-03-15

**Authors:** Karen O’Brien

**Affiliations:** University of Oslo in Norway, Oslo, Norway

**Keywords:** Anthropocene, Human dimensions, Mindsets, Noosphere, Transformation

## Abstract

Research on global environmental change has transformed the way that we think about human-environment relationships and Earth system processes. The four *Ambio* articles highlighted in this 50th Anniversary Issue have influenced the cultural narrative on environmental change, highlighting concepts such as “resilience,” “coupled human and natural systems”, and the “Anthropocene.” In this peer response, I argue that global change research is still paying insufficient attention to *how* to deliberately transform systems and cultures to avoid the risks that science itself has warned us about. In particular, global change research has failed to adequately integrate the subjective realm of meaning making into both understanding and action. Although this has been an implicit subtext in global change research, it is time to fully integrate research from the social sciences and environmental humanities.

“Welcome to the Anthropocene.” The cover photo of the May 28, 2011 edition of *The Economist* portrayed the Earth as a technical structure covered by riveted steel plates in dull blue colors. The accompanying story informed readers that “Humans have changed the way the world works. Now they have to change the way they think about it, too” (The Economist 2011). This was a remarkable statement from a magazine considered an icon of neoliberal economic policy, and it seemed to suggest that an awareness of human-induced global environmental change had finally penetrated the world of business and economics. Ten years later, is there any real evidence of a fundamental shift in thinking that moves beyond business as usual and towards an equitable and sustainable world?

Yes and no. How we think about the way that the world works has changed dramatically over the past decades. The years leading up to *The Economist* cover story saw an impressive amount of research on global environmental change that has transformed the way that we think about human-environment relationships and Earth system processes. The concept of the Anthropocene has contributed to a new way of describing the significant role of humans in shaping the Earth’s geology and ecology. Yet climate change, biodiversity loss, poverty, inequality, and other global problems are even more serious concerns today, and the timeframe for taking actions to meet international commitments is shrinking, increasing the risk of reaching “tipping points” and experiencing catastrophic losses (IPCC 2018; IPBES 2019). Business as usual has been proceeding at breakneck speed, interrupted only by the COVID-19 pandemic. This is an important moment to reflect on *how* conscious transformations to sustainability can be realized.

In this peer reflection, I start by acknowledging the contributions of global change research to a dynamic, interconnected view of the world. With specific reference to four key articles published in *Ambio*, it is clear that concepts such as “resilience,” “coupled human and natural systems,” and “the Anthropocene” represent important advances that have influenced both scientific and cultural narratives on environmental change. However, I would also argue that global change research is still paying insufficient attention to how to deliberately transform systems and cultures to avoid the risk of what Steffen et al. (2011, p. 14) describe as “the collapse of large segments of the human population or of globalised contemporary society as whole.” In particular, global change research has failed to adequately integrate the subjective realm of meaning making into both understanding and action. Not just meaning making in general, but the differences in and dynamics of meaning making, including how they relate to beliefs, values, agency, empowerment, creativity, emotions, and not the least, political action. A deeper approach recognizes the limits of what Berzonsky and Moser (2017, p. 16) refer to as the “’dominant social paradigm’ characterized by fragmentation, either/or thinking, an isolation of humans from nature, and a split of the material from the spiritual, the individual from community.” Without attention to the “deeper” human dimensions of global environmental change, it is likely that large-scale societal transformations will remain wishful thinking, rather than experienced realities.

The importance of meaning making has long been an implicit subtext within global change research. In fact, the realm of human thought and ideas, also referred to as the “noosphere,” has historically had a close relationship with understandings of ecology and geology (see Samson and Pitt 1999). However, even though this relationship has been recognized for over one hundred years, the dynamic aspects of meaning making have not been fully integrated into global change research, with exceptions such as research on climate change beliefs within cognitive psychology and on indigenous attitudes towards nature in anthropology and human geography. As an abstract representation of the subjective, interior world of individual and collective meaning making, the noosphere may be a useful starting point for inquiries into how humans relate to each other, to nature, and to the future. More important, it may provide insights into how this does (or does not) change over time and within different social and cultural contexts, such that we can better understand and promote rapid transformations to sustainability.

The four *Ambio* articles reviewed here include subtle yet significant references to the noosphere. For example, in “Resilience and Sustainable Development: Building Adaptive Capacity in a World of Transformations,” Folke et al. (2002, p. 437) called for “awareness of the need for a worldwide fundamental change in thinking and in practice of environmental management.” Emphasizing concepts of resilience and adaptive management, they highlighted the dynamic and non-linear nature of social-ecological change and the potential for irreversible regime shifts. They also considered the policy implications of resilience within the context of sustainable development, drawing attention to interrelationships between the biosphere and prosperous development of society, as well as the need for flexible and innovative collaboration. The article offered suggestions for how to operationalize sustainability, including by strengthening the perception of interdependence of humanity and nature and recognizing that “The outdated perception of humanity as decoupled from, and in control of, nature is an underlying cause of society’s vulnerability” (Folke et al. 2002, p. 438). This article touched on the relationship between thoughts and practices, which together with the call for a shift in perceptions, hints at the importance of meaning making, beliefs, and worldviews. This was important, as the paper summarized a report that fed into the 2002 World Summit on Sustainable Development in Johannesburg.

In “Coupled Human and Natural Systems,” Liu et al. (2007) emphasized the complexity of organizational, spatial, and temporal relationships, and highlighted the ways that cumulative and evolving impacts of past interactions influence current and future conditions. In describing coupled human and natural systems (CHANS), they pointed to the global yet heterogeneous nature of spatial interactions and the time lags between human decisions and their environmental effects, all of which complicate understandings and management strategies. Unprecedented rapid changes and tighter couplings at multiple scales were presented as an interdisciplinary challenge that called for integrated tools and assessments to produce “more ‘usable’ knowledge for sustainable ecological and socioeconomic benefits” (Liu et al. 2007, p. 646). In reflecting on the implications for management, governance, and policy, Liu et al. (2007) recognized that hubris in human attitudes toward natural systems was an impediment for progress. At the same time, they acknowledged that humans are not sufficiently represented in ecological science. The paper thus indirectly recognized the importance of subjective attitudes and meaning making and the need for a larger role for the social sciences and humanities in global change research.

In the same issue of *Ambio*, the article by Steffen et al. (2007) on “The Anthropocene: Are Humans Now Overwhelming the Great Forces of Nature?” described a profound shift in human-nature relationships. Placing the understanding of coupled human and natural systems within a wider historical context, the paper stressed that humanity is pushing the Earth into a state of *terra incognita*. The authors drew attention to some of the worst-case scenarios, noting that prior to the Anthropocene, humans “did not have the numbers, social and economic organization, or technologies needed to equal or dominate the great forces of Nature in magnitude or rate” (Steffen et al. 2007, p. 615). With the onset of industrialization in the 19^th^ century, humans transformed the environment at a global scale, as evidenced by dramatic rises in atmospheric concentrations of greenhouse gas emissions. In describing The Great Acceleration that started in 1945, Steffen et al. (2007) noted that the intellectual, cultural, political, and legal context at the time paid little attention to the impacts on Earth System processes. To ensure sustainability of the planet, they emphasized the need for a more reflective approach to development, noting that “Humanity is, in one way or another, becoming a self-conscious, active agent in the operation of its own life support system” (Steffen et al. 2007, p. 619). The very idea that part of the system is becoming self-aware of its impact on the system suggested a shift in meaning-making, which is a reflection that resonates with some interpretations of the noosphere (Samson and Pitt 1999).

Finally, the article by Steffen et al. (2011) highlighted the potential for managing the global environment in a more sustainable manner, and called for a fundamental change in our relationship to the planet we inhabit. In “The Anthropocene: From Global Change to Planetary Stewardship,” the authors reiterated some of the historical drivers and indicators of the Anthropocene, and pointed to the importance of biodiversity in maintaining sustainable environmental conditions. Importantly, they stressed that “We are the first generation with the knowledge of how our activities influence the Earth System, and thus the first generation with the power and the responsibility to change our relationship with the planet” (Steffen et al. 2011, p. 759). This article too hinted that changing our relationship with the planet involves more than behavioral shifts; it involves a shift in meaning-making that is expressed through actions that replace exploitative or controlling systems with ones that reflect a mindset of interdependence and stewardship.

These four *Ambio* articles identified the need for transformative change, and thus can be considered foundations for today’s rapidly-growing literature on transformations to sustainability. For example, Folke et al. (2002, p. 437) recognized that “humans can transform ecosystems into more or less desirable conditions.” Steffen et al. (2007) concluded that a business-as-usual approach will be insufficient to meet the challenges of the twenty-first century, and Liu et al. (2007, p. 644) recognized that “traditional development strategies need to be altered, and transforming them into sustainable practices is urgent…”. Finally, Steffen et al. (2011, p. 753) sounded a warning against ‘‘fiddling at the edges’’ and acknowledge that “[m]ore transformational approaches may be required.” The transformative approaches described by Steffen et al. (2007) ranged from geo-engineering or the deliberate manipulation of Earth system processes to strategies to reduce or modify human influence by adopting a “Planetary Boundaries” approach. Yet geoengineering is widely considered a continuation of business as usual, in that it does nothing to challenge the current political, economic, or cultural systems that drive environmental change, nor the paradigms and practices that maintain them. This suggests a rather limited vision for transformative responses within the biophysical discourse on global environmental change (Leichenko and O’Brien 2019).

The articles have certainly helped to steer global change research in a more integrated and action-oriented direction. For example, Folke et al.’s (2002) focus on sudden and abrupt changes can be considered a precursor to research on planetary boundaries and tipping points, which is now being applied to the concept of social tipping points (Bentley et al. 2014; Milkoreit et al. 2018; Otto et al. 2020). Liu et al. (2007) called for more attention to emergent properties, reciprocal effects, nonlinearity, and surprises in management and planning. In discussing the increased scale and pace of human-nature interactions, they used the example of diseases such as SARS that spread much faster than earlier due to globalization processes. Despite these insights, the recent COVID-19 pandemic reveals a massive failure to integrate knowledge and action. Steffen et al. (2011) remind us that a failure to act introduces the possibility for collapse, or the uncontrolled decline of a society or civilization.

What these articles did not address was *how* deliberate transformations to sustainability come about, particularly how transformations in perceptions, meaning making, and relationships with nature actually can and do shift, and how such changes play out in the political sphere. Importantly, in recent years there has been a dramatic increase in the number of research programs, projects, and articles on transformations to sustainability. Most of these are located within the social sciences and environmental humanities, and draw attention to the importance of integrating more complex understandings of social systems and more nuanced interpretations of human relationships with the natural world. In *Urgency in the Anthropocene*, Lynch and Veland (2018, p. 1) contend that the notion of the Anthropocene belongs to a modern European mythology and its linear view of time, emphasizing that “our narration of causation and expectation fundamentally determines the preparation for, response to, and recovery from each perceived manifestation of anthropogenic global change.” For example, representation of the Anthropocene as a “rupture” or deviation from Holocene conditions can be contrasted with an interpretation that highlights the “entanglement” of humans and other beings and processes in the Earth system (Harrington 2020). Indeed, more and more researchers are focusing on how to transform these entangled relationships, paying attention to the role of mindsets, meaning making, imagination, and narratives (Göpel 2016; Milkoreit 2017; Hochachka 2019).

There are also many critical and emancipatory approaches in the social science that acknowledge the ways that social structures and institutions can limit or expand the potential for humans and non-human species to flourish in the Anthropocene (Wright 2013). For example, pointing to the need to go beyond a general focus on “megatrends” and “humanity,” Brand (2016, p. 515) emphasizes a political ecology perspective, where “[w]hat is being examined is not ‘the environment’, the ‘environmental space’, ‘planetary boundaries’, or even the overuse of resources, ecosystems and sinks. Of interest are rather the capitalist, imperial and patriarchal forms of the appropriation of nature: i.e., the forms in which such basic societal needs as food and housing, mobility and communications, and health and reproduction are satisfied.” This focus on power, politics, gender, colonization, global inequality, and interspecies relationships has led to alternative interpretations of the Anthropocene, introducing terms such as the Manthropocene, the Capitalocene and the Chthulucene (Gibson-Graham 2011; Castree 2015; Haraway 2016).

Returning to the “Welcome to the Anthropocene” article in *The Economist*, one cannot help but notice that the “world of transformations” described by Folke et al. (2002) translated into the idea that planetary resilience “will probably involve a few dramatic changes and a lot of fiddling” (The Economist 2011), or more specifically geoengineering and technical innovations. Hmm. It is clear that transformations to an equitable, just, and thriving world will require more than this. Perhaps the key to a sustainable future lies not in just working to change the way that “others” think about the world, but to be alert and wary of the potential for hubris in the science of global change. The imperative for transformative change demands reflexive, strategic, inclusive, and diverse responses; integrating the noosphere into understandings of Earth System processes may help us not only to make sense of the current crises, but also to transform them.
